# Patientensicherheit in der endoprothetischen Weiterbildung: Führt die Weiterbildung von Operateuren in der primären Hüftgelenkendoprothetik in zertifizierten Endoprothesenzentren zu vermehrten Komplikationen?

**DOI:** 10.1007/s00132-021-04110-y

**Published:** 2021-04-30

**Authors:** S. Rohe, S. Brodt, C. Windisch, G. Matziolis, S. Böhle

**Affiliations:** 1 Waldklinken Eisenberg, Campus für Orthopädie, Medizinische Fakultät, Friedrich-Schiller-Universität Jena, Klosterlausnitzer Str. 81, 07607 Eisenberg, Deutschland; 2 Abteilung für Orthopädie und Unfallchirurgie, Helios Klinikum Blankenhain, Wirthstr. 5, 99444 Blankenhain, Deutschland

**Keywords:** Krankenhausaufenthaltsdauer, Chirurgische Ausbildung, Intraoperativer Blutverlust, Operationszeit, Hüfttotalendoprothese, Length of stay, Surgical education, Surgical blood loss, Surgical time, Total hip replacement

## Abstract

**Hintergrund:**

Ziel der Studie war die Untersuchung, ob sich das perioperative Outcome und operationsspezifische Prozessgrößen bei Patienten mit Hüftgelenksersatz signifikant verschlechtern, wenn die Operation durch einen unerfahrenen Operateur, assistiert durch einen erfahrenen Operateur, im Kontext eines zertifizierten Endoprothesenzentrums durchgeführt wird.

**Material und Methoden:**

Es wurden 1480 Patienten eingeschlossen, die zwischen 2013 und 2016 bei primärer Koxarthrose mit einer primären zementfreien Hüfttotalendoprothese (HTEP) versorgt wurden. Die relevanten Daten wurden retrospektiv aus dem Krankenhausinformationssystem, dem Entlassungsbrief und aus dem EndoCert-Bogen dokumentiert. Die Operateure wurden nach ihrer Qualifikation in erfahrene (Senior, > 50 HTEP pro Jahr) und unerfahrene Operateure (Junior, < 50 HTEP pro Jahr) unterteilt. Anschließend erfolgte der Vergleich der erhobenen Daten anhand dieser Unterteilung.

**Resultate:**

Es zeigte sich bei unerfahrenen Operateuren eine signifikante Verlängerung der Operationsdauer um 20,7 min (Senior 62,6 ± 20,4 min; Junior 83,3 ± 19,5 min; *p* ≤ 0,001), der Krankenhausverweildauer um 0,25 Tage (Senior 8,8 ± 0,9 d; Junior 9,0 ± 0,9 d; *p* ≤ 0,001) und eine Erhöhung der Transfusionshäufigkeit von Erythrozytenkonzentraten (Senior 0,6 ± 1,1 Stk.; Junior 0,9 ± 1,4 Stk.; *p* ≤ 0,001). Dagegen zeigte sich kein Unterschied bei perioperativen Komplikationen (*p* = 0,682) und beim perioperativen Blutverlust (Senior 1,3 ± 0,5 l; Junior 1,3 ± 0,5 l; *p* = 0,097). Zwischen Operationsdauer und Blutverlust bestand allerdings eine positive Korrelation (Senior r = 0,183; Junior r = 0,214; jeweils *p* ≤ 0,01).

**Schlussfolgerung:**

Die Ausbildung von unerfahrenen Operateuren an einem zertifizierten Endoprothesenzentrum führt bei Assistenz durch erfahrene Operateure nicht zur Reduktion der Patientensicherheit mit vermehrten Komplikationen. Aufgrund der Operationszeitverlängerung kommt es allerdings zu einer Mehrbelastung der Kliniken im Wettbewerb mit nichtausbildenden Kliniken, die nicht im DRG-System abgebildet ist.

## Abstract (en)

**Background:**

The aim of the study was to investigate whether the perioperative outcome and the operation-specific process variables in patients with total hip arthroplasty (THA) significantly deteriorate when the operation is performed by an inexperienced surgeon assisted by an experienced one in the context of a certified endoprosthesis center.

**Material and methods:**

1480 patients who received primary THA for primary coxarthrosis between 2013 and 2016 were included. The relevant data were retrospectively documented from the hospital information system, the discharge letter and the EndoCert form. The surgeons were divided according to their qualifications into experienced (senior surgeon, > 50 THA per year) and inexperienced surgeons (junior surgeon, < 50 THA per year). The collected data and measured variables were then compared based on this subdivision.

**Results:**

Inexperienced surgeons showed a significant increase in the duration of the operation by 20.7 min (senior 62.6 ± 20.4 min; junior 83.3 ± 19.5 min; *p* ≤ 0.001), as well as the length of hospital stay by 0.25 days (senior 8.8 ± 0.9 days; junior 9.0 ± 0.9 days; *p* ≤ 0.001). The frequency of transfusions of red cell concentrates was significantly increased with inexperienced surgeons (senior 0.6 ± 1.1 items; junior 0.9 ± 1.4 items; *p* ≤ 0.001). In contrast, there was no difference in perioperative complications (*p* = 0.682) or in perioperative blood loss (senior 1.3 ± 0.5 l; junior 1.3 ± 0.5 l; *p* = 0.097). However, there was a positive correlation between the duration of the operation and blood loss (senior r = 0.183; junior r = 0.214; each *p* ≤ 0.01).

**Conclusion:**

The training of inexperienced surgeons at a certified endoprosthesis center does not lead to a reduction in patient safety or increased complications when inexperienced surgeons are assisted by experienced surgeons. Due to the extended operating time, however, there is an additional burden on the clinics in competition with non-training clinics, which is not mapped in the DRG system.

## Einführung

Die endoprothetische Versorgung der fortgeschrittenen Hüftgelenksarthrose wurde 2007 in *Lancet* als der erfolgreichste Eingriff des 20. Jahrhunderts bezeichnet [1]. Im Jahr 2019 wurden in Deutschland laut statistischem Bundesamt 243.477 [2] und laut Endoprothesenregister 157.681 primäre Hüftgelenktotalendoprothesen (HTEP), davon 78,4 % zementfrei, implantiert [3]. Sie stellt damit die sechsthäufigste stationäre Operation in Deutschland dar [2]. Aufgrund der alternden Bevölkerung mit konsekutiver Zunahme der Hüftgelenksarthrose ist mit einer weiteren Steigerung der Operationszahlen zu rechnen. In den USA wird diesbezüglich ein Anstieg von 2005 zu 2030 um 174 % erwartet [4].

Die Implantation einer primären HTEP stellt einen hochstandardisierten Eingriff dar. Die intra- und perioperativen Komplikationsraten sind gering, wobei hier vor allem Wundinfektionen, tiefe Beinvenenthrombosen, neurologische Defizite, Implantatmigrationen und Frakturen auftreten [5, 6]. Durch eine Zertifizierung als Endoprothesenzentrum im Rahmen des EndoCert-Konzeptes mit personenbezogenen Mindestmengen soll ein Qualitätsstandard gesichert und die Komplikationsrate weiterhin gering gehalten werden [7]. Der Nutzen dieser Zertifizierung wird kontrovers diskutiert. So konnten Weber et al. zeigen, dass eine Zertifizierung nicht unmittelbar zur Verbesserung der Ergebnisqualität, gemessen an aufgetretenen Komplikationen und Funktionsscores, führt [8, 9]. Im Gegensatz dazu zeigten Bergschmidt und Lewinski et al. eine Verbesserung dieser [10, 11]. Im Rahmen des EndoCert-Konzeptes werden u. a. Operateure nach jährlichen Mindestmengen in Haupt- und Senior-Hauptoperateure unterteilt. Dabei ist die geforderte jährliche Mindestmenge implantierter Prothesen für Senior-Hauptoperateure 100 und für Hauptoperateure 50 [7]. Im Rahmen der Ausbildung assistieren Haupt- und Senior-Hauptoperateure weiterhin nichterfahrenen Operateuren, i. d. R. Weiterbildungsassistenten und jungen Fachärzten, die weniger als 50 dieser Eingriffe jährlich durchführen. Inwieweit eine Operation durch nichterfahrene Operateure die Patientensicherheit beeinflusst, ist noch nicht abschließend geklärt. So beschrieben Judge et al. und andere Autoren eine erhöhte Komplikationswahrscheinlichkeit mit schlechteren Ergebnissen bei nichterfahrenen Operateuren und in Krankenhäusern mit geringen Fallmengen [12–16]. Zenk et al. konnten dagegen im Setting eines zertifizierten Endoprothetikzentrums keine signifikante Komplikationszunahme oder Score-Verschlechterung bei unerfahrenen Operateuren verzeichnen [17, 18]. Es ist nicht abschließend geklärt, ob in zertifizierten Endoprothesenzentren darüber hinaus ebenfalls Unterschiede in Prozess- und paraklinischen Parametern zwischen diesen Gruppen bestehen. Unterschiede in der Operationszeit, der Dauer des Krankenhausaufenthaltes sowie des Blutverlustes, der Transfusionshäufigkeit und der postoperativen CRP-Werte als potenzielles Zeichen der Invasivität bei gleichem operativem Zugang sind erwartbar [19]. Um diese Fragen genauer zu klären, untersuchten wir retrospektiv die Primärimplantationen von zementfreien Hüftendoprothesen im Zeitraum von Januar 2013 bis Dezember 2016 in einem Endoprothesenzentrum der Maximalversorgung.

## Patienten und Methoden

### Patientenstichprobe

Es wurden 1480 Patienten, die in einem zertifizierten Endoprothesenzentrum der Maximalversorgung eine zementfreie Hüftendoprothese bei primärer Koxarthrose (ICD-10 M16.x) erhielten, retrospektiv in die Studienpopulation eingeschlossen. Patienten mit sekundärer Koxarthrose, aktiven Entzündungen, Tumorerkrankungen, rheumatoider Arthritis und Patienten, die sich in den letzten 3 Monaten einer Operation unterzogen hatten, wurden nicht eingeschlossen. Die Operation fand in der Regel in Allgemeinnarkose und nach intravenöser Single-Shot-Gabe eines Antibiotikums statt. Die Implantation der Prothesen erfolgte nach Hausstandard über einen modifizierten transglutealen Zugang nach Bauer [20]. Die Nachbehandlung erfolgte bei allen Patienten nach hausinternem Standardschema, soweit keine Komplikationen auftraten. Postoperativ wurde ein Hüftkompressionsverband angelegt. Ab dem ersten postoperativen Tag begann die Mobilisation unter 50 % Teilbelastung im 3‑Punkt-Gang bis zur Entlassung.

### Methodik

Von allen eingeschlossenen Patienten wurden retrospektiv aus dem Krankenhausinformationssystem (Fa. AGFA Orbis, Berlin, Deutschland) folgende Daten erfasst: Gewicht, Größe, BMI, Geschlecht, Alter, Operationsdauer, Krankenhausverweildauer, ASA-Status (ASA-PS), präoperatives sowie maximales postoperatives CRP-Level (Tag 1–4), prä- und postoperativer Hämatokrit, perioperative Transfusionsrate von Erythrozytenkonzentraten und Komplikationsrate während des stationären Aufenthaltes. Bezüglich der Komplikationsrate wurden Frühinfekte, periprothetische Fissuren, Implantatmigrationen (> 2 mm im postoperativen Röntgenbild im Vergleich zur intraoperativen Bildgebung), Nervenläsionen, oberflächliche Wundheilungsstörungen und Hämatome, die eine operative Revision nach sich zogen, berücksichtigt. Der Blutverlust wurde mithilfe der Formel von Brecher, Monk und Goodnough und das Blutvolumen mittels Nadler-Formel berechnet [21, 22]. CRP und Hämatokrit wurden im krankenhauseigenen Labor bestimmt. Dabei wurde für die CRP-Bestimmung ein qualitativer visueller Latex-Agglutinationstest verwendet (Cobac C 6000/Modul 501, Fa. Roche, Grenzach-Wyhlen, Deutschland. Erfassungsgrenze 0,2 mg/l, Nachweisgrenze 0,3 mg/l).

### Statistische Analyse

Die Datenerhebung wurde mittels Microsoft Excel 365 (Fa. Microsoft, Redmond, WA, USA) und die statistische Analyse mittels des Statistikprogramms SPSS 27 (Fa. IBM, Armonk, NY, USA) durchgeführt. Als Analyseansatz wurden nichtparametrische Tests zweier unabhängiger Stichproben gewählt und ein Mann-Whitney-U-Test als geeignetes statistisches Analyseverfahren angewendet. Zur Korrelationsanalyse wurde der Pearson-Test angewendet. Das Signifikanzniveau betrug *p* < 0,05.

## Resultate

Es wurden 1480 Patienten eingeschlossen, darunter 620 Männer und 860 Frauen (Senior 56 % der Patienten weiblich; Junior 63 % der Patienten weiblich; *p* = 0,008). Bei allen Patienten wurde ein Schaft im „Zweymüller-Design“ verwendet (CLS Spotorno, Fa. Zimmer Biomet Deutschland GmbH, Freiburg). Davon wurden 1040 von Senior- (70,3 %) und 440 von Junior-Operateuren (29,7 %) versorgt. Das durchschnittliche Alter betrug 68,6 ± 9,3 Jahre (Senior 67,8 ± 9,7 Jahre; Junior 70,5 ± 8,0 Jahre; *p* ≤ 0,001), der durchschnittliche BMI betrug 29,0 ± 5,2 kg/m^2^ (min. 15,8 kg/m^2^, max. 56,2 kg/m^2^; Senior 29,0 ± 5,3 kg/m^2^; Junior 28,8 ± 4,8 kg/m^2^; *p* = 0,983). Bezüglich der operierten Seite (links 47 %; rechts 53 %; *p* = 0,385) und des ASA-PS (2,2 ± 0,5; *p* = 0,746) unterschieden sich die Untersuchungsgruppen ebenso nicht, wie im Hinblick auf die dokumentierten Vorerkrankungen nach Einteilung in die ICD-Subgruppen.

**Figure 1:**
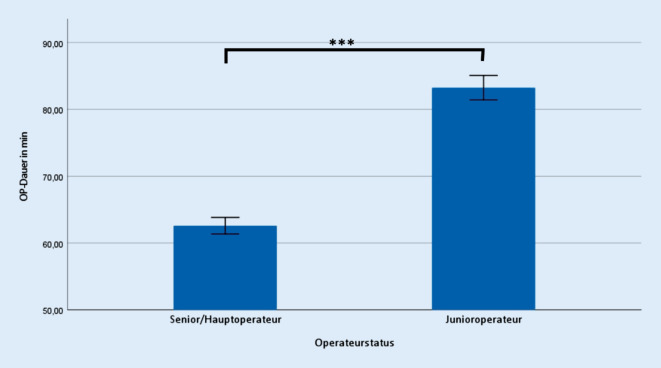

**Figure 2:**
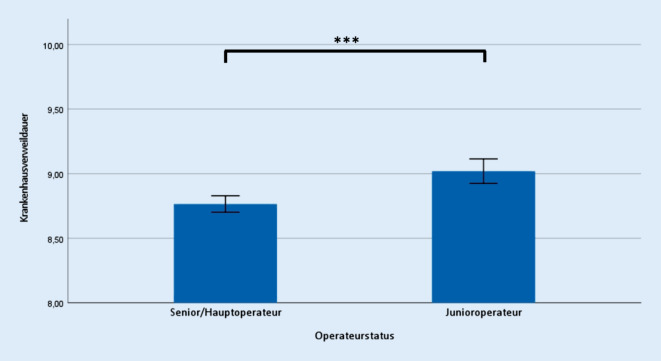

**Figure 3:**
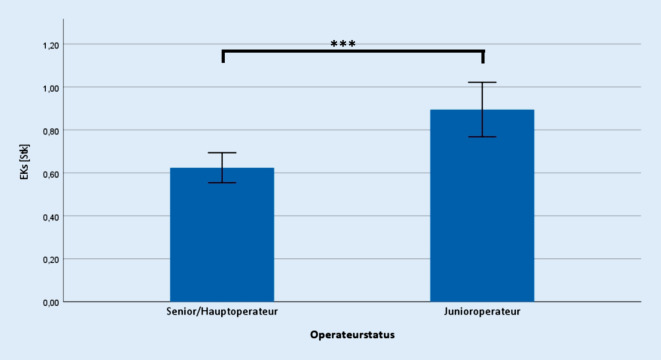

Bezüglich der erhoben operativen Daten zeigte sich ein signifikanter Unterschied hinsichtlich der Operationszeit (Senior 62,6 ± 20,4 min; Junior 83,3 ± 19,5 min; *p* ≤ 0,001) (Abb. 1), der Krankenhausverweildauer (Senior 8,8 ± 0,9 d; Junior 9,0 ± 0,9 d; *p* ≤ 0,001) (Abb. 2), sowie der Anzahl intra- und postoperativer Transfusionen von Erythrozytenkonzentraten (Senior 0,6 ± 1,1 Stk.; Junior 0,9 ± 1,4 Stk.; *p* ≤ 0,001) (Abb. 3).

**Figure 4:**
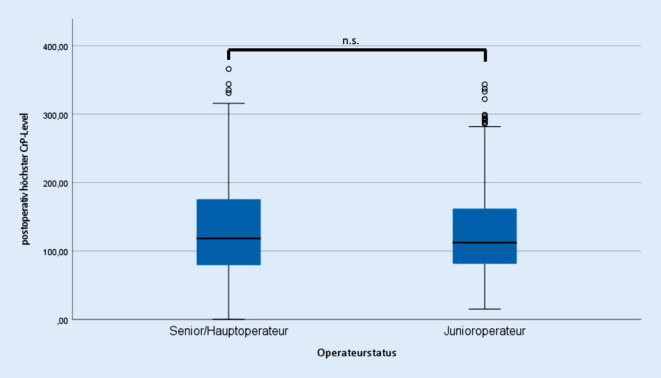

**Figure 5:**
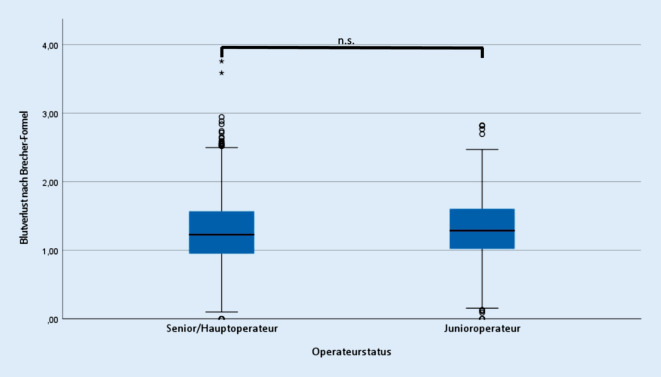

Die Komplikationen im Allgemeinen (*p* = 0,682) und differenziert entsprechend oben genannter Arten wiesen keine signifikanten Unterschiede zwischen beiden Gruppen auf (Tab. 1, dabei traten einzelne Komplikationen gehäuft bei denselben Patienten auf). Das präoperative CRP-Level unterschied sich ebenso wie das postoperative maximale CRP-Level nicht zwischen beiden Gruppen (Senior 129,5 ± 64,9 mg/l; Junior 127,0 ± 64,9 mg/l; *p* = 0,396) (Abb. 4). Bezüglich des intraoperativen Blutverlustes zeigten sich ebenso keine signifikanten Unterschiede (Senior 1,3 ± 0,5 l; Junior 1,3 ± 0,5 l; *p* = 0,097) (Abb. 5). Eine positive Pearson-Korrelation zwischen Operationsdauer und Blutverlust wurde dennoch in beiden Untersuchungsgruppen festgestellt (Senior r = 0,183; Junior r = 0,214; jeweils *p* ≤ 0,01).

**Table 1:** 

Komplikation	Senior		Junior		*p*
	*n*	%	*n*	%	
Revisionen	21	2,0	11	2,5	1,0
Thrombosen	5	0,5	1	0,2	1,0
Periprothetische Frühinfekte	10	1,0	3	0,7	1,0
Oberflächliche Wundinfekte	15	1,4	4	0,9	1,0
Hämatomausräumungen	5	0,5	2	0,5	1,0
Implantatmigration	5	0,5	4	0,9	1,0
Therapiebedürftige intraoperative Fissuren	14	1,3	5	1,1	1,0
Luxationen	1	0,1	1	0,2	1,0
Nervenläsionen (passager und chronisch)	7	0,7	7	1,6	1,0
Sepsis	2	0,2	0	0	–
Myokardinfarkt	3	0,3	0	0	–
Lungenarterienembolie	1	0,1	0	0	–
Gastrointestinale Infekte	2	0,2	0	0	–
Harnwegsinfekte	1	0,1	1	0,2	1,0

## Diskussion

In dieser retrospektiven Analyse konnte gezeigt werden, dass die Implantation einer zementfreien HTEP in der Ausbildung von Operateuren mit geringeren Fallmengen (weniger als 50 HTEP im Jahr) mit Assistenz durch einen erfahrenen Operateur in einem Endoprothesenzentrum nicht zu einer Reduktion der Patientensicherheit führt.

Im Gegensatz dazu beschrieben Koy et al. und andere Autoren eine erhöhte Komplikationsrate durch unerfahrene Operateure mit geringen Mindestmengen und Krankenhäuser mit geringen Fallzahlen [12–16], allerdings ohne Berücksichtigung eines prozessoptimierten Settings eines zertifizierten Endoprothesenzentrums und der Assistenz durch erfahrene Operateure. In unserer Analyse traten Komplikationen nicht gehäuft in einer der beiden Gruppen auf. Auch die Subgruppenanalyse der Komplikationen zeigte keine vermehrte Häufigkeit von einzelnen Komplikationen. Hier bestätigte sich die Sicherheit des Verfahrens, auch wenn die Operation durch einen unerfahrenen Operateur durchgeführt wurde, wie es bereits Zenk et al. zeigten [17]. Diese Sicherheit liegt vermutlich in einem bestehenden optimierten prozessorganisierten Umfeld und in der Assistenz durch einen erfahrenen Operateur begründet und unterstreicht, dass die assistive Begleitung jeder Operation durch einen erfahrenen Operateur sinnvoll ist. Prä- und postoperative CRP-Level als Marker für die Inflammation und potenziell für die Invasivität des Eingriffes bei gleichem operativem Zugangsweg zeigten ebenso keinen signifikanten Unterschied zwischen beiden Gruppen [19]. Vergleichsuntersuchungen aus anderen chirurgischen Gebieten bezüglich des Zugangstraumas zwischen erfahrenen und unerfahrenen Operateuren zeigten bei acht untersuchten Standardoperationen ebenso keinen signifikanten Unterschied in der 30-Tage-Mortalität [23].

Die Operationsdauer war bei unerfahrenen Operateuren signifikant um durchschnittlich 20,7 min verlängert. Zenk et al. beschrieben ebenfalls eine Zunahme der Operationsdauer bei unerfahrenen Operateuren. Hier lag die durchschnittliche Operationsdauer bei erfahrenen Operateuren (150–500 implantierte HTEP) bei 74,5 ± 25,5 min und bei unerfahrenen Operateuren (< 50 HTEP) bei 80,8 ± 21,9 min [18]. Dies ist durch korrigierende bzw. didaktische Eingriffe des anwesenden erfahrenen Operateurs, sowie über ein zu Beginn der Ausbildung eher zögerlicheres Operieren und häufigere Kontrollmechanismen aufgrund der mangelnden Routine zu erklären [24].

Trotz der verlängerten Operationszeit bei den unerfahrenen Operateuren fand sich kein signifikant erhöhter perioperativer Blutverlust im Vergleich zu erfahrenen Operateuren, obwohl in beiden Gruppen eine positive Korrelation zwischen Operationsdauer und Blutverlust bestand. Ein möglicher Erklärungsansatz ist die schon intraoperativ erfolgte Gabe von Erythrozytenkonzentraten, die in der Gruppe der unerfahrenen Operateure signifikant häufiger erfolgte und wodurch der für die Berechnung des perioperativen Blutverlusts benötigte postoperative Hämatokrit verbessert wurde. Dies stellt somit eine Limitierung dieser Studie dar. Ein weiterer Erklärungsansatz ist das signifikant höhere Alter und das häufigere weibliche Geschlecht der Patienten in der Gruppe der unerfahrenen Operateure. Alter und Geschlecht sind bekannte Risikofaktoren für intra- und postoperative Transfusionsbedürftigkeit und könnten letztlich in der Gruppe der unerfahrenen Operateure zu mehr Transfusionen und damit auch zu einer Maskierung des perioperativen Blutverlustes geführt haben [25]. Eine erhöhte Transfusionshäufigkeit bei unerfahrenen Operateuren bestätigen weiterhin die Ergebnisse einer australischen Studie [26]. Wilson et al. zeigten dabei ein 30 % höheres Risiko einer Transfusion bei Trainee-Operateuren im Vergleich zu Consultant-Operateuren, während das Risiko für medizinische, chirurgische und Wundkomplikationen ebenso nicht signifikant unterschiedlich war [26].

Die Krankenhausverweildauer war in unserer Untersuchungsgruppe der unerfahrenen Operateure um 0,25 Tage, wenn auch für die klinische Praxis eher unbedeutend, verlängert. Ein Einfluss der Erfahrung des Operateurs auf die Krankenhausaufenthaltsdauer wurde bereits beim Schulter- und Sprunggelenksersatz beschrieben [27, 28]. In der Übersichtsarbeit von Koy et al. wurde die Verweildauer bei zwei der elf Studien untersucht und zeigte ebenso einen positiven Effekt bei hohen personenbezogenen Fallzahlen [13]. In unserer Untersuchung könnte das signifikant erhöhte Alter der Patienten in der Gruppe der unerfahrenen Operateure (70,5 Jahre vs. 67,8 Jahre) eine Erklärung für den längeren Krankenhausaufenthalt darstellen, da die postoperative Mobilisation mit zunehmendem Alter häufig erschwert sein kann.

Unabhängig von der Auswirkung auf die Patientensicherheit führt eine verlängerte Operationszeit bei unerfahrenen Operateuren in Weiterbildungs- und Ausbildungskliniken jedoch auch zu einem vermehrten Ressourcenverbrauch. Beispielsweise resultierte in unserem Endoprothesenzentrum die Operationszeitverlängerung von durchschnittlich 20,7 min bei 440 von unerfahrenen Operateuren implantierten HTEP zu einer Mehrbelastung von 9108 Operationsminuten in 4 Jahren. Diese ist allerdings nicht im DRG-System abgebildet. Dadurch tragen Weiterbildungs- und Lehrkrankenhäuser eine erhebliche Mehrbelastung im Wettbewerb gegenüber den Krankenhäusern, die keinen Beitrag zur operativen Ausbildung leisten. Dies zeigte ebenso Windisch et al. in Bezug auf die Implantation von Kniegelenksendoprothesen [29].

Limitationen der Studie sind das monozentrische Design sowie die retrospektive Datenanalyse.

### Schlussfolgerung

Zusammenfassend zeigt sich, dass trotz Ausbildung eines unerfahrenen Operateurs die Patientensicherheit in einem zertifizierten Endoprothesenzentrum bei verlängerter Operationszeit und diskret erhöhter Transfusionswahrscheinlichkeit nicht gefährdet wird und die Ausbildung der zukünftigen Operateure nicht zu Lasten der Patienten geht. Allerdings wird deutlich, dass eine erhöhte Operationszeit, bei ca. 240.000 primären HTEP in Deutschland, einen immensen Ressourcenverbrauch in Ausbildungs- und Weiterbildungskliniken darstellt. Daher stellt dessen zukünftige Abbildung in einem anzupassenden DRG-System eine wichtige Stellschraube dar, um die Ausbildung auch weiterhin für Kliniken attraktiv zu machen, und um einen qualitativ hochwertigen Nachwuchs an Operateuren sicherzustellen.

## Fazit für die Praxis

- Es gibt keine Unterschiede zwischen erfahrenen und in der Weiterbildung befindlichen Operateuren in der Komplikationsrate nach Hüfttotalendoprothesen(HTEP)-Implantation bei Assistenz durch einen erfahrenen Operateur.
- Es bestehen signifikante Unterschiede für Operationsdauer, Dauer des stationären Aufenthaltes und Transfusion von Erythrozytenkonzentraten zwischen erfahrenen und unerfahrenen Operateuren.
- Unerfahrene Operateure benötigten durchschnittlich 20 min länger für eine HTEP-Implantation als erfahrene Operateure.
- Die operative Weiterbildung im Rahmen eines Endoprothetikzentrums findet nicht zu Lasten der Patienten statt, sondern auf Kosten der Klinik.
- Die Weiterbildungs- und Lehrkrankenhäuser tragen dadurch eine erhebliche Mehrbelastung im Wettbewerb mit Krankenhäusern, die keinen Beitrag zur operativen Ausbildung der Weiterbildungsassistenten leisten.

## Abkürzungen

ASA: American Society of Anesthesiologists
BMI: Body-Mass-Index
CRP: C‑reaktives Protein
DRG: „Diagnosis Related Groups“
EK: Erythrozytenkonzentrate
HTEP: Hüfttotalendoprothese
ICD: International Statistical Classification of Diseases and Related Health Problems
